# Durability Considerations in Replacing Blast Furnace Slag with Low-Grade Calcined Clay and Natural Pozzolan in Quaternary Cements

**DOI:** 10.3390/ma18215048

**Published:** 2025-11-05

**Authors:** Juan Manuel Etcheverry, Laurent Detemmerman, Krist Degezelle, Vadim Grigorjev, Laurena De Brabandere, Nele De Belie

**Affiliations:** 1 Magnel-Vandepitte Laboratory for Structural Engineering and Building Materials, Ghent University, 9052 Ghent, Belgium; 2 DEVAGRO, Beton and Recyclage Harelbeke, 8530 Harelbeke, Belgium

**Keywords:** calcined clay, lava, natural pozzolan, reactivity assessment, concrete durability

## Abstract

Belgium and the EU-27 face a shortage of suitable supplementary cementitious materials (SCMs) capable of supporting high levels of Portland cement substitution. To reduce CO_2_ emissions from the cement industry, blended cements incorporating low-grade calcined clay, limestone, and lava (a natural pozzolan) are investigated. Calcined clay is combined with limestone to produce a limestone–calcined clay cement (LC3). The reactivity of these new blends is assessed using isothermal calorimetry and compared to a reference blend with ground-granulated blast-furnace slag (GGBFS). Results show that mixtures with calcined clay develop slightly lower 28-day strength than those with GGBFS, while blends with lava exhibit strength gains only at later ages due to delayed pozzolanic activity. Overall, concrete made with low-grade calcined clay and lava achieves comparable compressive strength to the reference (CEM III/A), but with higher capillary porosity, leading to increased water absorption, drying shrinkage, and reduced freeze–thaw resistance. Despite these durability limitations, the sustainability assessment reveals that the LC3 mix with low-grade clay and lava has a lower global warming potential per unit strength at 28 days than CEM III/A and is competitive with CEM III/B.

## 1. Introduction

The production of Portland cement (PC) is responsible for a large part of global carbon dioxide emissions, estimated between 6% and 10% [1,2]. Given the expected rise in demand for concrete, there is a considerable need for alternative supplementary cementitious materials (SCMs) with local availability. The replacement of PC with SCMs is an important part of the CEMBUREAU plan to decarbonise the cement industry by 2050 [3,4]. SCMs reduce emissions by partially replacing PC, as they are typically not produced at the same high temperature of PC (some of them do not even require thermal activation) and because there is no limestone decomposition, the main process during which CO_2_ is released. A well-established and frequently used SCM is ground-granulated blast-furnace slag (GGBFS). However, the supply of GGBFS itself is expected to be significantly reduced in the near future due to changes in the steel production process; therefore, it is unable to meet the large demand of the cement industry. The amount of slag available is currently 5–10% of the amount of cement produced worldwide [5]. GGBFS is a valuable SCM (with a comparable price to PC) widely regarded as environmentally friendly in the cement industry, largely due to its favourable allocation methods (i.e., environmental burdens are mostly attached to steel as the main product because of the classification of the slag as an industrial by-product). Therefore, local cement and concrete producers are constantly in search of alternative SCMs that can replace GGBFS without significantly increasing the PC content in concrete mixes (maintaining a similar SCM content as in CEM III cements, while keeping strength development and durability performance).

An existing alternative is limestone–calcined clay cement (LC3). Previous research found that by combining limestone (LL) with calcined clay (CC), up to 50% of PC can be replaced without compromising performance [5]. In addition, there is enough proof that the development of a microstructure is not limited by the availability of portlandite [6]. Usually, clays with a kaolinite content in the range 40–60% are required as a raw resource which are then calcined around 650–800 °C to produce reactive calcined clays [7]. The properties of the CC depend mostly on the kaolinite content of the raw clay, with the fineness being the second most important parameter [8]. During the calcination process, kaolinite transforms into metakaolin. This metakaolin is mainly responsible for reactivity and in combination with PC and limestone, it is able to react within the first week, allowing the LC3 mixture to reach a similar strength to PC mixes. Non-kaolinitic clay phases such as illite or montmorillonite are also reactive after calcination but to a lesser degree compared to kaolinite and require broader calcination temperatures to reach dihydroxylation (500–900 °C) [5,9,10].

A different SCM that has recently received attention is recycled concrete fines. The crushing of concrete debris is often conducted with the aim of re-using the coarse aggregates in recycled aggregate concrete (RAC) and the recycled concrete powder is generally the last fraction to be repurposed [11]. Natural pozzolans, such as lava (LV), can also be used as an SCM and have the advantage of being locally available, similarly to clay, and thus able to partially sustain the large demand for cement [12]. Secondly, no additional heat is required for the activation of the material. However, natural pozzolans tend to possess only a limited reactivity and the pozzolanic activity is often only noticeable after an extended curing period [3].

The replacement of GGBFS with less reactive SCMs (usually containing less calcium) does not only affect the mechanical strength of concrete but also the durability characteristics. The reactivity of the SCM influences the development of the pore structure. When comparing blended cements containing GGBFS with traditional PC mixes, a lower connectivity of the pore network tends to be pointed out as beneficial for the durability characteristics of concrete. The ingress of water (carrying harmful substances) is part of almost every deleterious process in concrete structures [13]. In addition, water transport is key for freeze–thaw resistance. Concrete may deteriorate by the freezing of water inside saturated pores. Especially for clays with a kaolinite content above 40%, a significant pore refinement (in comparison to PC) has been reported, which is expected to contribute to freeze–thaw resistance. Previous studies showed that concrete mixes made with calcined clay containing sufficient kaolinite result in an improved freeze–thaw resistance comparable to that of concrete with GGBFS [14,15]. In contrast, clays containing large fractions of illite and montmorillonite have been reported to increase capillary porosity [9,13], consistent with lower reactivity, rather than kaolinite-type clays. Therefore, the kaolinite content of the calcined clay is not only important for the mechanical strength but also for the durability of concrete [16].

This study investigates the reactivity of an LC3 blend using a low-grade CC in combination with minor additions of the SCMs available in Benelux as potential replacement for CEM III/A. Lava is incorporated with the aim of enhancing the long-term performance of the blended cement containing low-grade calcined clays. Furthermore, naturally carbonated recycled fines (from stockpiles) are used, which are unlikely to have any chemical reactivity [17]. However, they can reduce the extraction of raw limestone, since they contain a high fraction of CaCO_3_. A small decrease in workability (in comparison to natural limestone filler) can be acceptable if it allows for the re-utilization of waste material. Isothermal calorimetry is used to determine the reactivity of the blended cements within the first days of hydration and to optimize the sulphate content of ternary and quaternary cement blends. Compressive strength tests on mortar samples, along with drying and autogenous shrinkage assessments, are used to identify the most promising mix for scaling up to concrete and conducting durability tests. Quaternary blends containing PC, LL, calcined clay, and lava are assessed for porosity [18], pore connectivity, shrinkage, and freeze–thaw resistance with de-icing salts. The results are compared to those of a reference mix containing GGBFS. In summary, this study aims to replace CEM III/A while maintaining strength development and to assess properties that could potentially compromise durability in non-structural concrete field applications.

## 2. Materials and Methods

### 2.1. Materials

#### 2.1.1. Cementitious Materials

The reference cement blend (CEM III/A according to EN 197-1) is prepared with 50% GGBFS and 50% PC (CEM I 52.5 N). The limestone is a commercial high-purity CaCO_3_ filler. The calcined clay is sourced from a local quarry and calcined in the laboratory at 750 °C for 4 h. Thermogravimetric analysis (TGA) reveals a kaolinite content around 20% (Figure S1 in the Supporting Information), which classifies CC as a low-grade calcined clay. The lava is a volcanic material originating from the Eifel region. The lava is commercially available as a 0–4 mm fraction. Recycled concrete fines are sourced from the waste fraction of the concrete debris crushing process. The fine fraction (0–2 mm) is obtained as a secondary product of the process. Spraying with a ~1 wt.% phenolphthalein-ethanol solution reveals that the recycled fines are carbonated, consistent with long-term exposure (from several months to years) to the environment in stockpiles.

The CC, lava (LV), and recycled fines (F) are milled in the laboratory to reach a similar fineness to PC (see particle size distribution (PSD), D_v10_, D_v50_, and D_v90_ in Table 1). The particle size distribution (PSD) is determined using an Occhio FC200 M-HR optical particle analyser, Occhio s.a., Angleur, Belgium, which determines particle sizes in the range of 200 nm to 800 µm. The chemical composition is determined with X-ray fluorescence spectrometry (XRF), Malvern Panalytical, Malvern, UK. The loss on ignition (LOI) obtained for LL is utilized to convert the CaO in CaCO_3_. The particle density is determined using the Le Chatelier flask according to ASTM C188. The fractiles of the PSD (D_v,10_, D_v,50_, and D_v,90_), the oxide composition, and the particle density of the materials are reported in Table 1. The full particle size distribution curves can be observed in the Supporting Information File (Figure S2).

#### 2.1.2. Blended Cements

Six different blended cements are initially prepared to investigate the effectiveness of the SCMs in the mixes. The blended cements are made using different amounts of CC, lava, recycled fines, and limestone, as well as gypsum. Binder formulations are reported in Table 2. An in-house-made CEM III/A with 50% GGBFS is used as the main reference. A mix incorporating 45% GGBFS, 10% limestone filler, and 45% PC is also included to clarify the effect of partially substituting GGBFS with LL in paste and mortar experiments. Paste specimens are prepared as described in Section 2.2.2. Mortar prisms with the six different blended cements (Table 2) are prepared according to EN 196-1, using a sand-to-binder ratio of 3:1 and a w/b ratio of 0.50.

#### 2.1.3. Concrete Mixes

Two batches of concrete samples are made, one with the CEM III/A and another with the 25CC-10LL-15LV. The latter quaternary blended cement (concrete) is designated as a CEM II/C-M according to EN 197-5. The CEM III/A concrete with 50% GGBFS is used as a reference. The mix design of both concrete types is given in Table 3. The binder content is the same in both concrete mixtures, 340 kg per m^3^, and the concrete is made with 100% coarse recycled aggregates (RAs) with BENOR certification. The water absorption after 24 h of the RAs is 4.9%. To compensate for the absorption, additional water is added to the mixture. The flow and the air content of the fresh concrete is measured according to EN 12350-5 and EN 12350-7, resulting in a similar flow and air content for both mixtures.

### 2.2. Methods

#### 2.2.1. Water Demand

A standard consistency paste (SCP) test is performed to determine the influence of calcined clay and lava on the water demand of the mixtures. The necessary w/b ratio for a standard consistency paste is determined according to ASTM C187. Three mixtures (Table 4) are tested, all including 65% PC and 10% limestone. The remaining 25% is either quartz (as reference), CC, or lava. These ratios are chosen to have a similar SCM replacement ratio to the LC3 system in Table 2.

The SCP test shows a noticeable difference in the mixing water necessary to reach a standard consistency paste. The mixtures with quartz, lava, and calcined clay need a w/b ratio of 0.27, 0.24, and 0.35, respectively. Thus, calcined clay increases the water demand considerably, whereas lava keeps the water demand constant or even slightly lower compared to the mix containing quartz. This means that lava may increase workability. The reduced workability of mixtures with CC must be compensated with either additional water or a superplasticizer addition. In this study, the water-to-binder (w/b) ratio is always kept constant and differences in workability between the different mixtures are compensated by superplasticizer addition.

#### 2.2.2. Isothermal Calorimetry

Isothermal calorimetry is conducted at 40 °C using a TAM Air calorimeter, TA Instruments, New Castle, DE, USA. All dry materials and mixing water are accurately weighed and preconditioned in an oven at 40 °C for 24 h before testing. The paste mixes are prepared by adding the demineralised water (w/b = 0.40) and superplasticizer to the mixtures with CC to achieve a sufficient flow. No superplasticizer is needed for the mixes containing GGBFS (Table 2). The paste is mixed at 1600 rpm for 2 min. Then, 14 g of paste is taken from the mixing container (70 g) using a plastic pipette and transferred to the glass vial. The lid is placed, and the container is lowered into the calorimeter, where the heat released is recorded for 4 days.

In addition to isothermal calorimetry on the novel binder mixes, a modified version of the R3 test method is used to determine the reactivity of the novel SCMs. The modified version of the R3 test [19] uses a potassium hydroxide solution (KOH) and calcium hydroxide (CH) (differing from the KOH, CH, and a potassium sulphate solution in ASTM C1897-20) and test temperature of 50 °C instead of 40 °C

#### 2.2.3. Mechanical Strength Tests

The mortar prisms described in Section 2.1 are demoulded after 24 h and cured in water (20 °C) until the required test age (2, 7, 28, 56, and 90 days) for the determination of the compressive strength.

The compressive strength of the concrete mixes (Table 3) is determined by casting 150 mm cubes according to EN 12390-2/3. The samples are cured in water (20 °C) until the required test age (2, 7, 28, 56, and 90 days) for the determination of the compressive strength.

#### 2.2.4. Durability Assessment

All concrete specimens are demoulded after 24 h and stored in a curing room (20 °C and >95% RH). The exceptions are the specimens used to measure the shrinkage, which, immediately after demoulding, are stored in a climate-controlled chamber at 20 °C and 60% RH.

The samples for porosity and the capillary imbibition tests (10 cm diameter and 5 cm height) are sawn from larger cylinders (10 cm diameter and 20 cm height). Top and bottom surfaces (2.5 cm height) are discarded. Curing time for all concrete samples for durability is 60 days in total.

#### 2.2.5. Capillary Imbibition

Three cylindrical samples (5 cm in height and 10 cm in diameter) are tested for capillary imbibition for each concrete type. Samples are cured at 95% RH and 20 °C for 60 days and preconditioned by placing them in an oven at 40 °C until the difference in the mass over 24 h is lower than 0.1%. Immediately after, the samples are placed in plastic bags for three days to enable the samples to reach a homogeneous moisture distribution (IRAM 1871:2021). The sides of the specimens are wrapped with aluminum-butyl tape (waterproof) to ensure unidirectional water flow during capillary imbibition. The level of water in contact with the specimens is always in between 1 and 3 mm. The mass of the samples is recorded after 15 min, 30 min, 1 h, 2 h, 3 h, 4 h, and 6 h. Next, the specimens are weighed every 24 h for one week and weekly up to 56 days.

#### 2.2.6. Porosity

First, three cylindrical samples (5 cm in height and 10 cm in diameter) are put into a vacuum chamber for 2 h when the water is allowed to enter the container while maintaining the vacuum. Next, the samples are left inside the water-filled tank for 24 h. Then, the samples are weighed in open air (M_a_) and under water (M_w_). The capillary porosity (Equation (1)) and open porosity (Equation (2)) are determined by drying the samples at 40 °C (M_d40_) or 105 °C (M_d105_), until a mass difference over a period of 24 h of less than 0.1%.

(1) $$CP\;\left(\%\right)=\frac{M_{a}-M_{d40}}{M_{a}-M_{w}}\;\cdot \;100$$

(2) $$OP\;\left(\%\right)=\frac{M_{a}-M_{d105}}{M_{a}-M_{w}}\;\cdot \;100$$

#### 2.2.7. Mercury Intrusion Porosimetry (MIP)

The porosity (MIP) is tested on concrete samples made with CEM III/A and CEM II/C-M. The hydration of the samples was stopped by using 2 cycles of solvent exchange (isopropanol) followed by 1 day of drying under light vacuum [20]. MIP was conducted using a Pascal 140/440 system (Thermo Fisher, Waltham, MA, USA).

Two grams of sample was placed in the dilatometer. To ensure complete filling of the intergranular voids, a vacuum was initially applied before allowing mercury to enter. A preliminary pressure of 200 kPa was then exerted in the low-pressure chamber to facilitate pre-intrusion. The dilatometer was subsequently transferred to the high-pressure unit, where the pressure was progressively increased up to 400 MPa while recording the intrusion curve. For pore entry size calculations, a mercury contact angle of 142° and a surface tension value of 0.48 N/m were used.

#### 2.2.8. Shrinkage

Shrinkage is measured on mortar samples (Table 2) and concrete samples (Table 3). The size of the testing specimens is 4 × 4 × 16 cm^3^ and 10 × 10 × 40 cm^3^, respectively. The initial shrinkage measurements are taken immediately after demoulding the prisms, (24 h after casting). Samples for autogenous shrinkage are wrapped in aluminum tape immediately after demoulding. Autogenous and total shrinkage are measured in accordance with EN 12390-16, with the exception that the mortar prisms are cast in moulds containing metallic pins on the extremes which allows us to monitor the change in length using a digital comparator (accuracy 1 µm) instead of DEMEC gauge points. The change in length for concrete prisms is measured using DEMEC gauge points glued on the surfaces of the prisms, as defined by EN 12390-16.

#### 2.2.9. Freeze–Thaw Resistance

The freeze–thaw resistance of concrete is tested using cylindrical concrete samples (10 cm in diameter and 5 cm in height). The test and the preparations are performed in accordance with EN 12390-9 after a curing age of 60 days, as previously described. A 3 mm layer of 3% NaCl solution is put on the top surface (sawn samples) to include the influence of de-icing salts. The scaling damage is weighed after 7, 14, 28, 42, and 56 freeze–thaw cycles. Each cycle consists of a temperature variation from 22 °C to −15 °C (measured via a thermocouple on the surface of a dummy specimen with a layer of water and de-icing salts inside the freeze–thaw chamber.

#### 2.2.10. Environmental Impact Calculations

The ISO 14040/44 2006 methodology is utilized for the life cycle assessment (LCA) of the concrete mixes. Although replacing PC with SCMs reduces the global warming potential (GWP) of the mixes, the reactivity of the SCMs as well as their production process should be considered in the calculations. An environmental performance metric is utilized to assess the environmental impact of the various formulations, taking into account their mechanical efficiency. This metric evaluates the CO_2_ emissions per m^3^ of concrete to deliver 1 MPa of strength.

The CO_2_ indicator is calculated for a cubic metre of concrete (cradle-to-gate), which involves all required intermediate treatments for (recycled) aggregates, calcined clays, limestone, PC, lava, and GGBFS. The impacts are regionalized using the current electricity and fuel mixes in Belgium, as detailed by [21]. The RA is considered waste material, with no allocation of environmental impacts to its generation. CO_2_ burdens for primary crushing operations, classifying operations, and a secondary crushing are assigned [22]. A transportation distance of 100 km is considered (processing and distribution). Although the treatment and CO_2_ emission of the SCMs may vary with location, availability of raw materials, and production processes, this study adopted the following: calcined clay and limestone production values from Pillai et al. [23] and the PC and GGBFS from [24], as listed in Table 5. The blast furnace slag is a by-product of steel production, and the basic intermediate treatment to produce the GGBFS includes granulation, drying, and grinding operations. An economic allocation procedure based on a previous work of some of the authors is applied [24], in which 0.84% of the impacts related to the steelmaking process are allocated to GGBFS production [25]. The burdens for extraction, primary crushing, classifying, and milling operations are allocated, with a 200 km transport distance considered for lava. Transportation is assumed to occur by truck (0.015 kg CO_2_ eq/kg lava), aligning with the fact that 80% of cement transport within the EU-27 is carried out by truck [26].

## 3. Results and Discussion

### 3.1. Isothermal Calorimetry

#### 3.1.1. Modified R3 Test on SCMs

The results of the modified R3 test on the anhydrous calcined clay, lava, and inert quartz are reported in Table 6, providing an initial indication of the reactivity of the CC and lava.

According to the classification (modified R3 test) proposed by [19], the CC is classified as pozzolanic with mid reactivity. The lava is situated on the boundary between inert and low reactivity materials. Compared to CC, lava has a higher heat release between 7 and 10 days which relates to its higher late degree of pozzolanic activity. In terms of pozzolanic activity, CH consumption for lava is much lower than for CC.

In summary, the CC not only reacts much faster, but it is also more reactive than the lava. CC is unable to achieve the reactivity of GGBFS, which is approximately 450 J/g SCM after 10 days at 50 °C [19], this seems to be associated with low kaolinite content, which is about 20%. Table 6 shows the cumulative heat release (after 3, 7, and 10 days) and CH consumption following the modified R3 test. The CH consumption is measured via thermogravimetric analysis and the consumption of portlandite from quartz mixes is used as a reference to calculate the extra consumption of CH due to the pozzolanic activity of CC and lava at 10 days.

#### 3.1.2. Blended Cements

**Figure 1:**
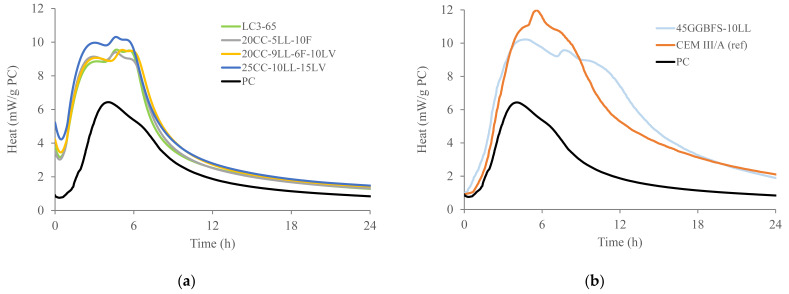

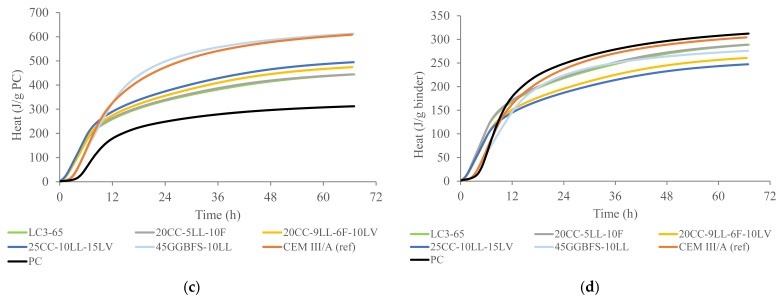
(**a**,**b**) Heat flow (mW/g PC), (**c**) cumulative heat (J/g PC), and (**d**) cumulative heat (J/g binder) for the PC and blended systems from Table 2.

Figure 1a–c display the heat flow and total heat curves for the different binders obtained by isothermal calorimetry (40 °C) and are normalized per gram of PC. An initial induction period, followed by the alite dissolution peak, is observed in Figure 1a. The alite dissolution peak is also often indicated as a C(-A)-S-H and CH precipitation peak [27]. This peak is usually followed by a peak or hump associated with the formation of secondary ettringite [27], also often referred to as an aluminate peak. Figure 1a shows that the hydration of alite is significantly enhanced for all blended systems (compared to PC) because of the filler effect provided by the SCMs. Please note that, in this study, all SCMs (except the coarsest fraction of the calcined clays) are finer than PC (Table 1). Figure 1b shows that the addition of GGBFS appears to have a minor (but still measurable) effect in the duration of the main peak. In any case, the finesses of the SCMs and the clinker contents are much more important factors, as explained by [27].

Concerning the aluminates peak, mixes containing calcined clays (Figure 1a) have limited differences in the sulphate depletion point (valley in between alite and secondary ettringite peak). A third peak or hump is identified in Figure 1a,b, which are accelerated in comparison to previous studies [27,28] due to testing conditions at 40 °C instead of 20 °C, as previously explained by some authors [24,29]. For the 45GGBFS-10LL system (Figure 1b), the addition of limestone (in comparison to CEM III/A), resulted in a slightly delayed AFm peak (as well as the sulphate depletion point) due to limestone being slightly coarser than GGBFS. Note that the same amount of sulphate was used for both mixes.

Figure 1c shows the cumulative heat during the first 3 days of hydration (approximately equivalent to 7 days at 20 °C) for all the systems, in which the lowest heat/g of PC is for the PC system, followed by the calcined clay containing mixes, and then GGBFS mixes. These values are in agreement with the reactivity observed in the R3 test, which revealed that GGBFS is more reactive at early ages than both the low-grade calcined clays and the lava.

When similar data are plotted in J/g binder (Figure 1d), it can be observed that LC3-65 and CEM III/A mixes can achieve a similar heat release during the first days of hydration simply because of the 15% extra PC added in LC3-65 versus CEM III/A. Despite a limited contribution of the lava which seems to be observed at early ages (expected according to the late pozzolanic reactivity observed via the R3 test), a contribution of the lava within the first 28 days is expected, which cannot be measured via calorimetry so is clarified via compressive strength measurements in next section.

Figure 1d shows the cumulative heat (J/g binder) release during the first days of hydration measured via isothermal calorimetry, providing a clear indication that the early development of microstructures can be slightly slower for the calcined clay-containing systems at a similar clinker content than for the CEM III/A reference. It is worth mentioning that these differences (50 J/g binder during the first week of hydration) can be considered rather minor when the final goal is to produce concrete mixes with a similar concrete strength class at 28 days [28].

### 3.2. Mechanical Strength

#### 3.2.1. Mortars Compressive Strength

**Figure 2:**
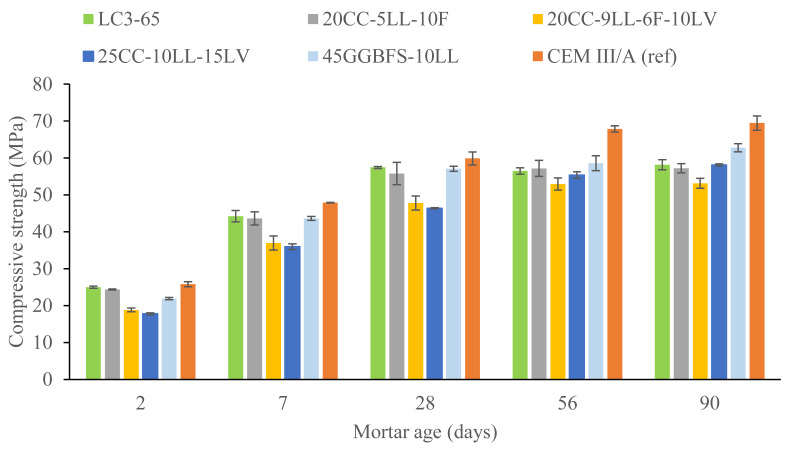
Compressive strength evolution of standardized mortar mixes made with the binders in Table 2.

Figure 2 displays the compressive strength development for standardized mortar samples made with the binders in Table 2. The development of the compressive strength (2–28 days of hydration) of mortar mixes with low-grade calcined clay and 65% PC (LC3-65 and 20CC-5LL-10F) is similar to the reference mix CEM III/A and 45GGBFS-10LL. However, to achieve similar strength development in low-grade CC-containing systems, the proportion of PC in these mixes needs to be higher than in CEM III/A, with a required PC content of 65% compared to 50%. The two mixtures with low-grade calcined clay and lava (20CC-9LL-6F-10LV and 25CC-10LL-15LV) have the lowest compressive strength up to 56 days, when all the systems appear to reach a strength in the order of 55–60 MPa (except for CEM III/A which reaches a higher strength), consistent with the low reactivity of the lava at early ages (see Table 6).

In this study, the limestone–calcined clay cement blends were only compared to a CEM III/A in terms of strength because that is the material that we expect to replace. In any case, CEM III/A tends to have a similar early age heat release (at 40 °C) to PC (Figure 1d). The two mixtures, LC3-65 and 20CC-5LL-10F, were found to be able to reach a similar strength development as a CEM III/A within the first 28 days of hydration. Nevertheless, they reached a plateau from 28 to 90 days The most likely explanation for this plateau is the low kaolinite content of the raw clay locally available; 40 to 60% MK content is the optimum for LC3 technology. The mix containing increasing contents of lava and clay (25CC-10LL-15LV) was able to increase the compressive strength beyond 28 days, reaching the same strength as the LC3-65 and 45GGBFS-10LL mixes. This indicates that, despite the metakaolin content of the local clays being lower than the optimum for LC3 production, low-grade clays are still a valuable source for fast-reacting SCMs [14,30] and it may be feasible to use a low-grade limestone–calcined clay ‘doped’ with late reacting pozzolanic material (in this case lava) as an alternative binder.

The 20CC-5LL-10F mixture with recycled fines yields a similar performance to its respective counterpart mix (LC3-65) without fines, similarly to the isothermal calorimetry results discussed in the previous section. Thus, the results suggest that it is possible to (partially) substitute raw limestone with naturally carbonated recycled fines as an alternative source of calcium carbonate, which is mostly interesting from a circularity perspective as it decreases the need for natural resources and it avoids the downcycling of naturally carbonated recycled fines (currently mostly used for soil stabilization). A recent study from some of the authors resulted in a similar outcome when adding recycled fines in concrete mixes with a high PC content [17]. Phase assemblage and microstructural evaluations would be necessary to clarify the underlaying mechanisms that resulted in the similar strength development observed among several systems in this section.

**Figure 3:**
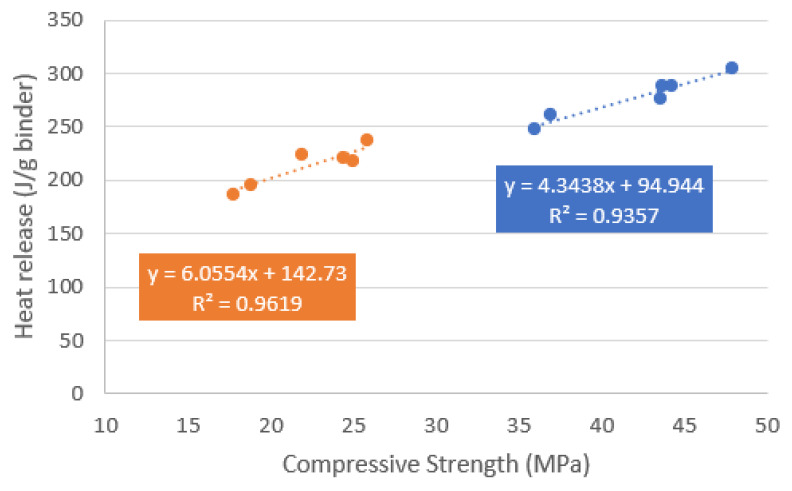
Correlation between heat release (paste scale) and compressive strength (mortar mixes).

Overall, the early compressive strength of the mortars is in line with the isothermal calorimetry results. The correlation between the cumulative heat release at 24 h and the mortar compressive strength at 2 days is plotted in Figure 3 (orange) as well as between the cumulative heat release at 48 h and the mortar compressive strength at 7 days (blue). The slope of both trend lines is similar, linking the heat release to the strength development process.

The decent mechanical performance of 25CC-10LL-15LV (high enough to allow for demoulding at early ages), supported by the early chemical reactivity of the CC shows the potential use as a CEM III/A replacement without a significant drop in compressive strength at 90 days. Thus, this blended cement is further tested at concrete scale and further designated as CEM II/C-M. A mix with CEM III/A is utilized as the reference for testing the performance at the concrete scale.

#### 3.2.2. Concrete Compressive Strength

**Figure 4:**
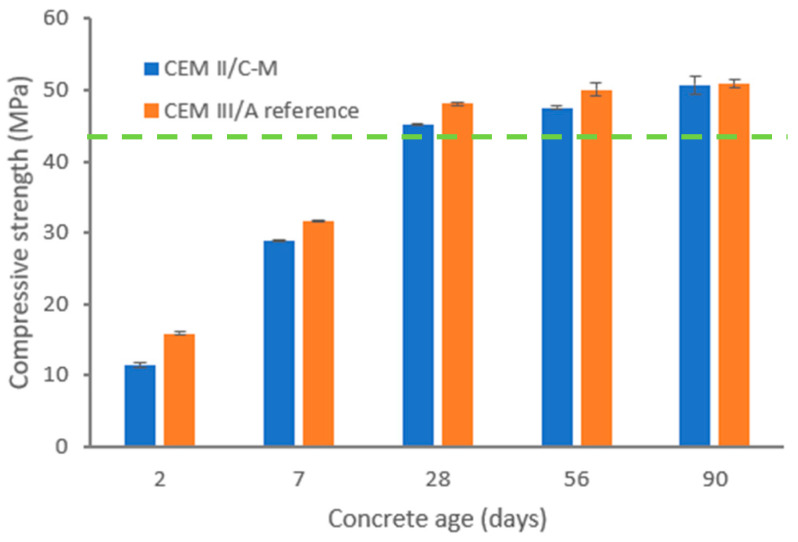
Compressive strength evolution of concrete mixes from Table 3. The 42.5 MPa cement strength class is indicated by the green dashed line.

The results of the concretes’ compressive strength are given in Figure 4. The early age strength of the CEM II/C-M is slightly lower than the CEM III/A reference, which is in agreement with previous discussions at the paste and mortar scale. The compressive strength of the concrete mixes (where the binder is ~10% in volumetric terms) is not only determined by the binder, but also by the granular skeleton, which has an important effect. This aspect explains that the differences in strength between CEM II/C-M and CEM III/A are less marked than for standardized mortar samples (binder represents ~17% in volumetric terms).

Both concrete types achieved the design’s compressive strength (28 days) of 42.5 MPa at concrete scale and a compressive strength at 90 days of 50 MPa. This shows that CC and likely LV continued forming hydration products beyond 28 days, whereas for GGBFS, the development of strength after 28 days is less pronounced.

### 3.3. Durability

#### 3.3.1. Porosity

**Figure 5:**
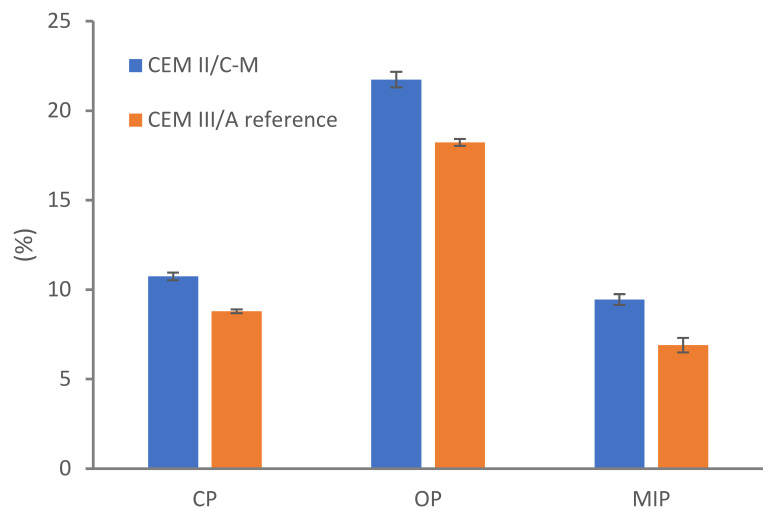
Capillary, open, and MIP porosity of concrete made with CEM III/A and CEM II/C-M.

The porosity measurements are presented in Figure 5. The capillary and open porosity of CEM II/C-M increase by 22% and 19%, respectively, compared to CEM III/A. MIP results confirm this trend, indicating a more porous cement matrix. Several factors may explain this increase:

(i) SCM reactivity and phase assemblage: the reactivity of the incorporated SCMs and the resulting phase assemblage are influenced by the reduction in calcium content. Compared to GGBFS, CC and LV contribute less calcium, affecting matrix densification [31].

(ii) The addition of up to 15% LL has been reported to enhance hydration in low-SCM systems, where calcium availability is not a limiting factor [6]. However, in PC blends with only 50% PC, replacing a reactive SCM (e.g., GGBFS) with LL can lead to a dilution effect, increasing porosity relative to the reference mix [5,13]. Note that in Figure 2, the 90 days compressive strength is affected by less than 10% (compare CEM III/A and 45GGBFS-10LL mixes).

(iii) Cement volume differences: due to the lower particle density of CC, a higher binder volume (per m^3^) could be obtained when dosed by mass (kg/m^3^), potentially increasing porosity. As discussed in the next section, this effect is expected to be limited.

#### 3.3.2. Capillary Imbibition and Water Absorption

**Figure 6:**
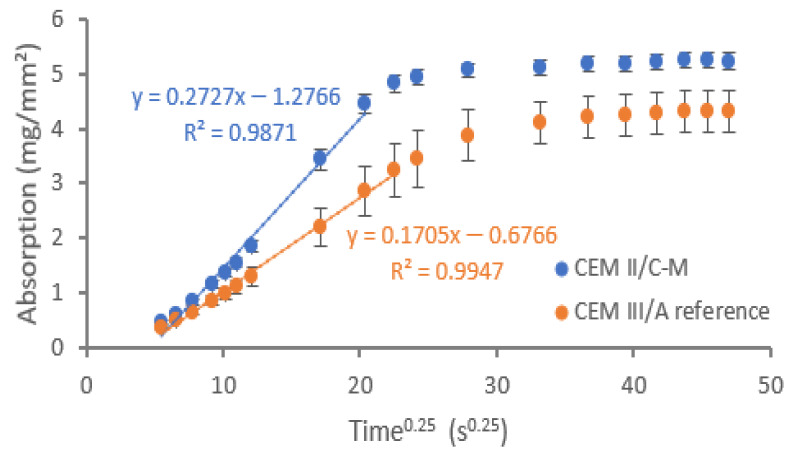
Capillary water uptake of the CEM II/C-M specimens and the CEM III/A references.

The capillary imbibition of the concrete specimens is plotted in Figure 6 as a function of the fourth root of time [32]. It is immediately noticeable that the capillary imbibition rate of the CEM II/C-M is higher than the reference mix. This is a logical consequence of the higher capillary porosity of this concrete type. Nevertheless, the increase in the primary capillary imbibition rate is 59%, which is remarkably higher than the increase in capillary porosity (22%).

The capillary imbibition rate mainly depends on the connectivity of the pores and the amount of capillary pores [33]. The addition of ‘low-reactive’ pozzolans tend to show a higher absorption and capillary porosity [9]. LC3 mixes made with illite and montmorillonite were also reported to increase sorptivity compared to LC3 with kaolinite clays. Since the transport properties of concrete mostly depend on the capillary water ingress, this increase in capillary imbibition rate might negatively influence the durability characteristics of the CEM II/C-M concrete in comparison to CEM III/A.

**Figure 7:**
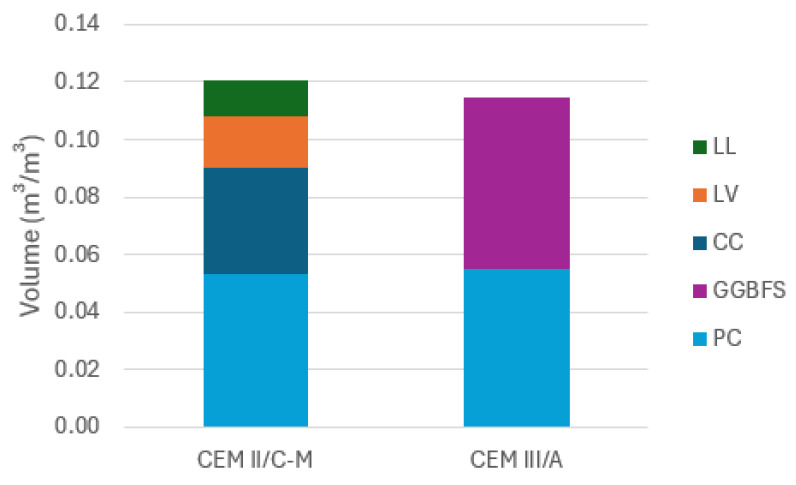
Binder volume for CEM II/C-M and CEM III/A calculated using the densities from Table 1.

The cement volume differences mentioned in Section 3.3.1 could also explain the increased capillary imbibition. Due to the fact that the CC has a low particle density, a larger paste volume is added when using dosages expressed in weight (kg/m^3^), leading to a larger volume of binder per m^3^ of concrete. If there is a higher volume of paste in the CEM II/C-M than in CEM III/A, then a higher capillary imbibition rate as well as a higher water absorption might be expected [34,35]. The difference in cement volume is calculated in Figure 7. These volumes are obtained using the density of the binders from Table 1. When comparing both cement volumes, the CEM II/C-M has a 0.5% higher volume of cement than the CEM III/A. Thus, this increase in binder content in volumetric terms can (only) partially explain the higher imbibition rate of the CEM II/C-M, but the higher connectivity of the capillary porosity is still deemed to be the dominant factor.

#### 3.3.3. Shrinkage

The incorporation of less reactive SCMs than GGBFS may result in a slower strength development, which can potentially lead to issues such as shrinkage cracking (tensile strength lower than tensile stress). The high water demand of calcined clays may exacerbate shrinkage-related cracking. This phenomenon is closely linked to the early age behaviour of the material, where increased evaporation combined with delayed hydration can cause shrinkage cracking or contribute to autogenous shrinkage under self-desiccation conditions [36]. In addition, the lower reactivity of these SCMs was reported to lead to a more open pore structure, particularly at early ages. A more porous matrix might result in higher drying rates and increased moisture loss, potentially accelerating drying shrinkage.

**Figure 8:**
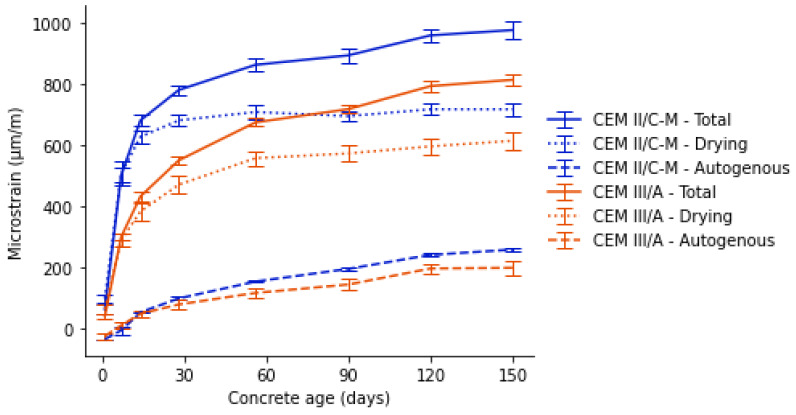
Total, drying, and autogenous shrinkage strain of concrete after 150 days.

**Figure 9:**
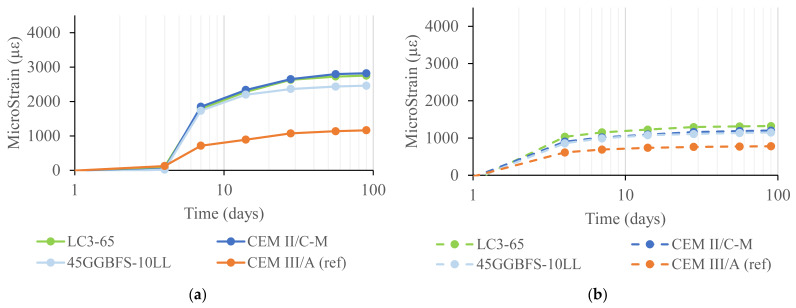
(**a**) Drying and (**b**) autogenous shrinkage strain of selected mortar mixes from Table 2.

Shrinkage measurements were performed on both mortar prisms and concrete prisms. The results of the concrete shrinkage strain given in Figure 8 show a higher drying shrinkage in the first 14 days for the CEM II/C-M. The drying shrinkage rate slows down for this concrete after 28 days compared to the CEM III/A. Autogenous shrinkage is comparable for both concrete types, especially within the first 28 days. Afterwards, a slightly higher autogenous shrinkage is noticed for the CEM II/C-M, consistent with the greater pozzolanic activity of clay and lava. This is in line with the findings at the mortar scale (Figure 9), where specimens with CC and limestone also showed a higher drying shrinkage and a moderately higher autogenous shrinkage compared to the reference mixtures with PC + GGBFS.

Results at the mortar scale (Figure 9a) confirmed that although calcined clay cement mixes shrink more than CEM III/A, such an effect is not necessarily linked to the differences in reactivity between the low-grade clay and GGBFS. Instead, it is the limestone addition that boosts the drying shrinkage. This is clear when the results of mixes CEM III/A and 45GGBFS-10LL are compared. There, the addition of 10% limestone yields shrinkage values in the same order as for all limestone–calcined clay-containing mixes in this study. In addition, the comparison of drying shrinkage between mixes LC3-65 and CEM II/C-M also confirms that the reduction in the PC content of the mixes does not necessarily yield higher shrinkage values (within the boundaries of these mixes; 65% PC in LC3-65 and 50% PC in CEM II/C-M). Figure 9b shows the autogenous shrinkage for the mortar mixes described and indicates that the effect of adding limestone is mostly seen within the first week, with all the mixes showing parallel trends after this timeframe.

The higher drying shrinkage strain, resulting in a larger total shrinkage, could also be explained by the higher accessible porosity of the CEM II/C-M. An increased connectivity of the pore network facilitates a more rapid evaporation of moisture [37]. The combination of higher early age shrinkage and lower early age strength might be seen as a greater risk of shrinkage cracking in the CEM II/C-M. However, comparing the micro-strain values of the concrete mixtures (Figure 8) with those of the mortar mixtures (Figure 9) reveals that shrinkage is not exclusively dependent on the binder mix design. In fact, the type of aggregates and the granular skeleton play a fundamental role—one that is even more significant than the binder itself—in shrinkage behaviour.

None of the materials tested in this study have shown signs of (micro)cracking due to shrinkage (visual inspection). Specific environmental conditions such as high temperature, excessive wind (both favouring greater drying rates), and/or very low temperature during casting (delaying hardening) must be considered as relevant parameter for on-site applications of either CEM III/A or CEM II/C-M mixes.

#### 3.3.4. Freeze–Thaw Resistance

The high quantity of non-kaolinitic phases in the locally available calcined clay is the main motivation for addressing the possible increase in scaling damage for which the failure mechanism is extensively discussed. This study does not aim to discuss the effect of salt (NaCl) concentration on the process. That is because of three reasons, (i) two concrete types are tested using the same type and concentration of salt, (ii) NaCl crystallization itself (from a brine) cannot take place above −22 °C [38] (testing setup reaches −15 °C), and (iii) it is very well known that salt crystallization has a pessimum effect at 3% concentration, with greater or lower values than 3% resulting in a much lower amount of descaled material.

**Figure 10:**
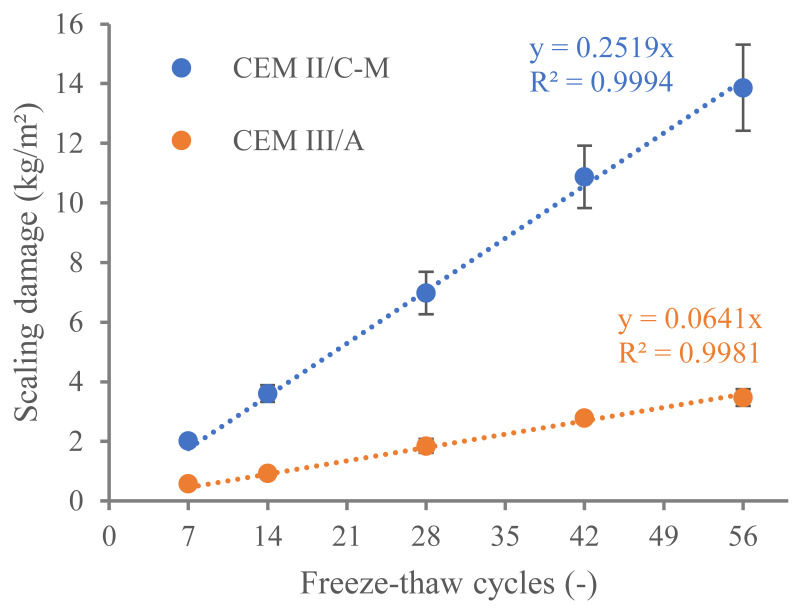
Cumulative scaling damage from 7 to 56 freeze–thaw cycles.

The freeze–thaw resistance of the two concrete types was tested with the inclusion of de-icing salts. The cumulative scaling damage after a certain number of freeze–thaw cycles is shown in Figure 10. The lower freeze–thaw resistance of the CEM II/C-M compared to the reference CEM III/A is evident. A constant trend is observed where the scaling damage of the CEM II/C-M is nearly four times higher than the CEM III/A. The linear trend observed for both concrete samples rules out the possible damage related to macrocracking of one of the binder types, which would lead to scaling damage with an exponential trendline [38]. To provide the reader with an order of magnitude, a maximum value of 1.0 kg/m^2^ is recommended by NBN EN 1339 2003 after 28 cycles of freeze–thaw with de-icing salts for paving blocks.

The freeze–thaw damage mechanisms (with de-icing salts) have been extensively studied for construction and building materials. The size of the pores appears to play a relevant role on the failure mechanism. Hydraulic pressure, crystallization pressure, and glue-spall mechanisms have been reported as possible failure mechanisms in buildings materials [39]. For hydraulic pressure, freeze–thaw resistance mainly depends on three aspects: degree of saturation (higher than ~90% [38]), the porosity, and pore connectivity. The damage occurs when pores are saturated with water and the water freezes. However, the freezing process occurs at different temperatures depending on the pore size, with greater pore diameters (>100 µm) freezing at temperatures (ΔT) near to 0 °C and nanopores (<10 nm) at temperatures in the range of −16 °C. These temperature values (ΔT) are calculated using the Gibbs–Thomson equation (Equation (3)) deducted in [39], assuming an ice front in an existing air void (with radius r) propagating into a cylindrical pore with radius r_p_. This expression assumes constant entropy for the liquid and the crystal within that temperature range [ΔS_fv_ = 1.12 × 10^6^ J/ (°C. m^3^)], a curvature K_cl_ = 2/r for the ice bulging in the pore, and r = r_p_ − δ, with δ (thickness of the unfrozen layer of water against the pore wall) equal to ~1.0 nm for cement paste [39].

(3) $$\left(r_{p}-\delta \right)≅-\frac{2\gamma_{CL}}{{\Delta S}_{fv}\Delta T}$$

Based on the previous equation, it can be noted that pores with a diameter lower than 6 nm (mainly gel pores), could not have a significant influence in the freeze–thaw damage using the aforementioned testing setup (for which the lowest temperature was −15 °C). This is consistent with the literature, indicating that water inside gel pores rarely freezes under these testing conditions [40].

According to hydraulic pressure, when the material starts to freeze (for instance from the outer part of the sample), small capillary pores act as an internal restriction for the water in bigger pores that, thermodynamically speaking, has to freeze first. This pressure is the result of the ice front pushing the water deeper in the sample (note that there is an increase in volume when water freezes and water has some viscosity (η), so that it will experience some resistance to this movement from the pore walls). There might be a local failure when this pressure created on the pore wall exceeds the tensile strength of the material. If the material is not close to its saturation degree, the presence of air will (partially) allow this pressure to be released.

Equation (4) expresses the flow rate (dv/dt) as a function of the gradient pressure (ΔP) on the cylindric pore (with radius R and length L).

(4) $$\frac{dv}{dt}=\Delta P\frac{\pi R^{4}}{8L}$$

This equation can be rearranged by considering dv/dt equal to the area of cylindrical pore multiplied by dx’/dt, where dx’ is to increase the movement of the waterfront deeper into the pore. Then, dx’ is equal to the advance of the ice front (dx) multiplied by (1 − ρ _Ice_/ρ _W_), with ρ being the density of ice and water, respectively. Therefore, Equation (5) can be obtained.

(5) ΔP=8ηLR21−ρiceρwaterdxdt

**Figure 11:**
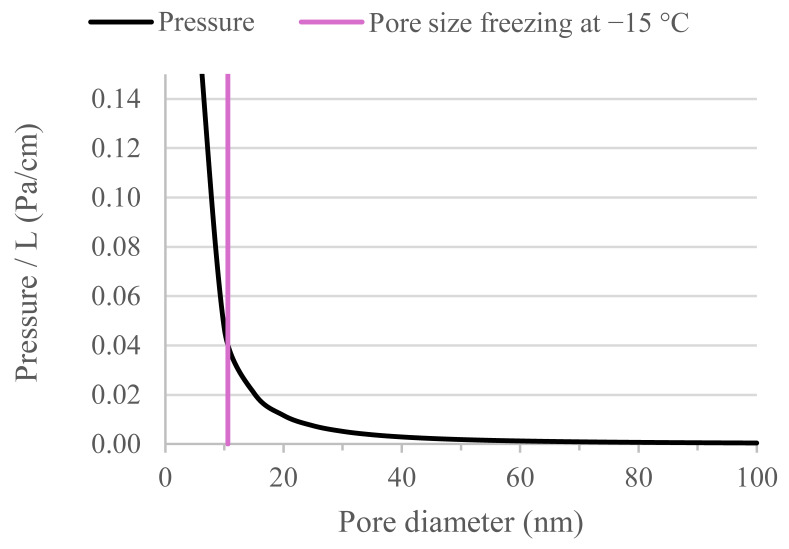
Pressure per unit of length as a function of pore diameter calculated using Equation (5).

By plotting Equation (5) for different pore diameters, Figure 11 is obtained. This figure reveals that the hydraulic pressure per unit length exponentially decreases with the increase in pore diameter.

An air void analysis could have been performed to identify if there are significant differences in the L (usually called air void spacing in aired intruded concrete) between the two mixes. However, this parameter was only reported significant with the addition of an air entraining agent that creates hydrophilic pores acting as pressure sinks [41]. Figure S3 in the Supporting Information shows the freeze–thaw resistance of samples saturated under vacuum before the NaCl solution was added, aiming to fill any hydrophilic pores potentially created by the superplasticizer addition. Only very limited variations in freeze–thaw damage are observed. This indirectly suggests that differences in the spacing factor (L) between air voids are not the main reason for the fourfold increase in freeze–thaw damage associated with CEM II/C-M.

**Figure 12:**
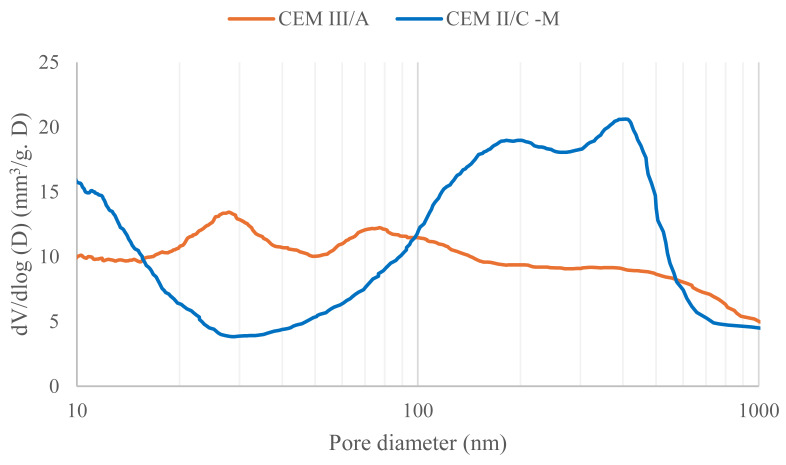
Pore size distribution of CEM III/A and CEM II/C-M obtained from MIP measurements.

Naturally, to estimate the influence of the net pressure exerted in the different mixes, the volumes of pores within specific pore ranges should be accounted for. From MIP analyses (and taking into account the possible limitations of the pore model used for MIP), it can be observed that concrete with CEM II/C-M is more porous (9.5%) than CEM III/A (6.9%) within the pore range of 100,000 to 4 nm. However, the porosity in the range 100 to 10 nm amounts for CEM II/C-M to ~2.2% while for CEM III/A, ~3.0% (Figure 12). If the same analysis is made in the range 30 to 10 nm (the most critical range of pores observed in Figure 11), the porosity of CEM II/C-M is ~1.2% while for CEM III/A it is ~1.5%.

This seems inconsistent with CEM II/C-M having a freeze–thaw resistance four times worse than CEM III/A, while both materials have similar mechanical properties. The same occurs when the threshold pore size is analyzed (in the order of 900 nm for concrete with CEM III/A and 700 nm for concrete with CEM II/C-M). To confirm this behaviour, samples made with the same binder type were tested following the exact same protocol (NBN EN 1339 2003) but adding water instead of the 3% NaCl solution (Figure S3). By doing so, it is possible to confirm that no damage (descaled material) is observed, even after 90 cycles of freeze–thaw. Therefore, hydraulic pressure cannot fully explain a freeze–thaw resistance four times worse for CEM II/C-M than for CEM III/A in this study.

Crystallization pressure and the so-called glue-spalling by [42] are other types of mechanisms that could induce the failure which appears to happen more often in site conditions and could occur even when the elements are not fully saturated [39].

**Figure 13:**
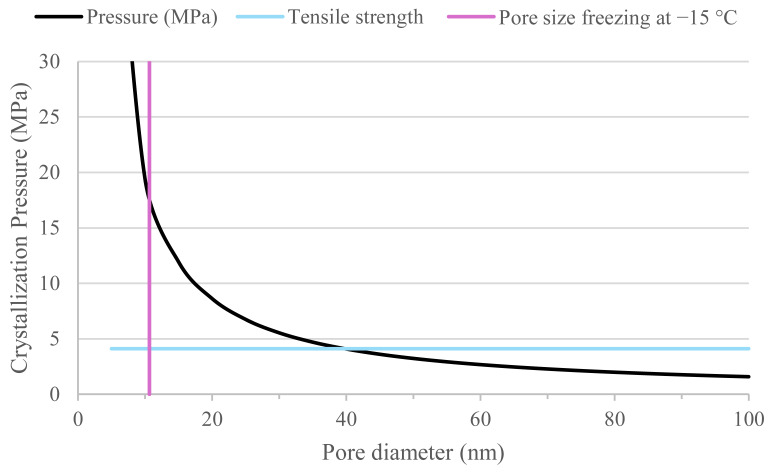
Ice crystallization pressure as a function of the pore diameter.

Crystallization pressure created by the propagation of ice in the pore structure is directly proportional to the variation in temperature plotted in Equation (3) and it is given by Equation (6).

(6) $$P≅-\frac{2\;\gamma_{CL}}{\;\left(r_{p}-\delta \right)}$$

where P is the local pressure (when the crystal of ice enters the capillary pore with radius r_p_, it sucks water from it, inducing a negative pressure). If this equation is plotted for pores with a radius between 5 nm and 50 nm (characteristic of cementitious materials), it can be observed that pores with a diameter greater than 40 nm (Figure 13) do not result in a local pressure that exceeds the overall tensile strength of our materials (approximated to be 10% of the compressive strength value [39,43]). It can be seen here that the smaller the pore radius, the higher the pressure. However, as previously explained, gel pores should not contain ice in the current testing conditions (−15 °C in CEN/TS 12390-9). As for hydraulic pressure, the difference in porosity within the 10–30 nm and 10–100 nm ranges between CEM II/C-M and CEM III/A does not account for the fourfold lower freeze–thaw resistance observed in CEM II/C-M. Please, note that it does not imply that there is no effect of crystallization pressure on the freeze–thaw damage. Instead, it suggests that crystallization pressure alone cannot be the main reason of the differences observed in the freeze–thaw damage (Figure 10).

The fact that the difference in scaling damage between the CEM II/C-M and the CEM III/A is a factor of four, whereas the capillary porosity is ‘only’ 22% higher and the capillary imbibition rate is 60% higher, initially led us to think that the most viable explanation for the inferior freeze–thaw resistance of the CEM II/C-M was an increase in total porosity and pore connectivity. This type of analysis is not uncommonly found in the literature of cementitious materials. However, if the difference in porosity was indeed what caused CEM II/C-M to perform four times worse than CEM III/A, the theoretical pressure could be roughly estimated by multiplying pressure values in Figure 13 and the number of pores in each pore range (Figure 12) and integrating such a curve. The integration of the curves should, in principle, yield a value at least four times higher for CEM II/C-M compared to CEM III/A. However, within the pore diameter range of 10 to 40 nm—where the individual pore pressure exceeds the local tensile strength (as shown in Figure 13)—the integration of both curves indicates that CEM II/C-M exhibits only about a 40% increase relative to CEM III/A.

The inconsistencies shown in this section indicate that the effect of hydraulic pressure and crystallization pressure would hardly explain the differences shown in Figure 10, implying that salt scaling (very likely glue-spall mechanism [38]) is most likely to explain the differences in freeze–thaw of the samples. This is clearly supported by the fact that samples tested at 0% NaCl (freeze–thaw cycles according to NBN EN 1339 2003 with water instead of NaCl solution) resulted in negligible descaled material for both concrete types.

Further research is needed to better understand the mechanisms driving the significant differences in scaling damage observed for CEM II/C-M and CEM III/A. Previous studies [15,44] have demonstrated that microcrack initiation and interfacial porosity strongly influence freeze–thaw deterioration. Therefore, the role of the interfacial transition zones and possible microcrack development must be investigated in future work. A future study will also assess whether the inclusion of entrained air and/or a reduction in the water-to-binder ratio can more effectively enhance the freeze–thaw resistance of CEM II/C-M cement incorporating low-grade calcined clays. Although the use of CEM II/C-M did not show an important decrease in the overall compressive strength, it could be because the dilution effect provided by the addition of limestone had a significant effect on the tensile strength or fatigue behaviour of the novel mix. A lower local tensile strength and/or fatigue resistance of the mixes, combined with the increased porosity and pore connectivity discussed in previous sections, are expected to directly influence the scaling resistance under the glue-spall mechanism.

#### 3.3.5. Global Warming Potential

**Figure 14:**
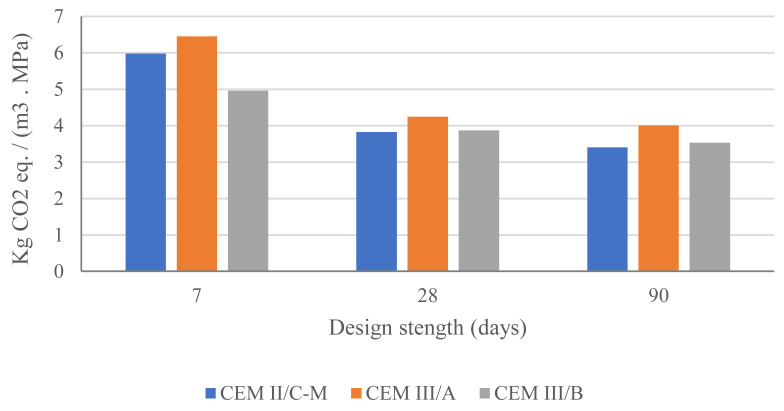
GWP per strength unit (7, 28, and 90 days) of 1 m^3^ of concrete for mixtures with CEM III/A, CEM II/C-M, and CEM III/B from Ref. [22].

The GWP per strength unit (at 7, 28, and 90 days) of 1 m^3^ of concrete for mixtures with CEM III/A and CEM II/C-M is shown in Figure 14. Additionally, a concrete made with CEM III/B from [22], using a similar granular skeleton and materials but with a reduced water-to-binder ratio (0.45 instead of 0.50) to achieve the 42.5 N strength class at 28 days, is included to illustrate the impact of higher GGBFS replacement factors. While reducing cement content decreases the GWP, it also reduces strength. Therefore, the coefficient shown in Figure 14 accounts for performance by considering GWP per unit of compressive strength (GWP/f’c).

A lower GWP/f’c coefficient indicates fewer CO_2_ emissions per unit of compressive strength. Figure 14 demonstrates that, at 7 days, CEM III/B (with a reduced water-to-binder ratio) is more favourable in terms of GWP/f’c compared to CEM II/C-M. However, when 28 days is used as the design parameter, as common in practice, CEM II/C-M emerges as the more convenient solution, a trend that persists at 90 days.

The analysis highlights that, although reducing cement content is a valid strategy for lowering GWP, the performance of supplementary materials in the mix must also be considered. For instance, while the production-scale GWP of CEM III/B is lower (164 kg CO_2_-eq/m^3^ compared to 173 kg CO_2_-eq/m^3^ for CEM II/C-M), the environmental-performance coefficient favours the CEM II/C-M mixture due to its superior strength development. Nevertheless, mixes with lower cement content than those studied here can still provide superior environmental alternatives when a lower strength class (e.g., 32.5 MPa) is sufficient for the application [31].

## 4. Conclusions

This study evaluated binder mixes based on LC3 blends with minor additions of pozzolanic lava and/or untreated recycled fines as replacements for CEM III/A. The experimental results showed that lava did not meaningfully increase early age strength; its contribution to mortar compressive strength became only slightly noticeable after 28 days, consistent with its late pozzolanic activity and limited reactivity shown by the reactivity test. Low-grade calcined clays contributed to strength development relatively quickly (within 28 days), but further strength gain after 28 days was limited at the mortar scale.

When calcined clays, limestone, and lava were used to replace GGBFS in mortar mixes, both drying and autogenous shrinkage increased relative to CEM III/A. However, mortars made with CEM III/A partially substituted with 10% limestone showed similar shrinkage values to CEM II/C-M, indicating that a small limestone addition may lead to comparable shrinkage behaviour.

At the concrete scale, the CEM II/C-M mix reached the design compressive strength of 42.5 MPa and matched the 90-day compressive strength of CEM III/A, although its early age compressive strength was slightly lower than that of CEM III/A. Durability testing revealed an increase in capillary and open porosity of about 20% for concrete made with CEM II/C-M compared to the CEM III/A reference. This higher porosity, together with a less favourable pore size distribution and possibly lower local tensile strength or fatigue resistance, corresponds with a significant increase in freeze–thaw scaling damage for CEM II/C-M.

Finally, the CEM II/C-M formulations delivered a lower GWP per unit f’c at both 28 and 90 days compared to CEM III/A. This shows that not only a successful replacement of GGBFS in terms of strength has been found but also the CEM II/C-M mix containing locally available calcined clays and pozzolanic lava resulted in a more efficient use of materials in the Flanders region. A future perspective is as follows: in specific (non-)reinforced applications within the Flanders region (excluding vibro-compressed elements such as small-size pavers, tiles, concrete brinks, or blocks), freeze–thaw resistance might be a critical performance parameter in horizontal surfaces able to accumulate water/brine after spreading de-icing salts (such as, paver flags, concrete slabs for sidewalks, or pavements). Future research will seek for recommendations to enhance the freeze–thaw resistance of CEM II/C-M mixtures while maintaining a low GWP/f’c coefficient in these elements. The carbonation of the material and the quality of the finished surface may influence the freeze–thaw damage of horizontal elements, and will also be assessed.

## Figures and Tables

**Table 1:** Physical properties and chemical composition of the binders.

	PC	CC	LV	F	LL	GGBFS
D_v,10_ (µm)	9.50	7.01	6.39	4.95	6.42	5.86
D_v,50_ (µm)	21.47	27.93	16.40	14.38	13.16	13.32
D_v,90_ (µm)	37.81	49.32	38.24	33.78	34.29	24.21
Density (g/cm^3^)	3.15	2.30	2.75	2.71	2.69	2.89
Chemical composition (%)						
CaO	73.20	10.78	17.39	46.22	-	40.25
Fe_2_O_3_	3.96	12.16	26.93	3.25	0.09	0.43
SiO_2_	12.69	36.23	28.99	36.30	0.39	30.48
Al_2_O_3_	3.79	9.44	7.01	5.91	0.13	12.18
MgO	1.45	2.28	3.71	2.17	0.50	8.13
SO_3_	2.17	0.16	0.16	2.01	-	0.08
K_2_O	0.24	1.52	2.80	0.50	-	0.45
Na_2_O	0.87	0.56	1.18	1.35	-	0.58
TiO_2_	1.56	1.15	2.73	2.21	-	-
MnO	0.06	0.02	0.20	0.06	-	0.25
CaCO_3_	-	-	-	-	98	-
LOI (%)					41.3	

**Table 2:** Binder formulations for the studied pastes and mortar systems (in wt%).

Mixture ID	PC	CC	LL	F	LV	GGBFS	Gypsum	Superplasticizer (mL/ 100 g PC)
LC3-65	65	23.5	11.5	-	-	-	3.50	0.20
20CC-5LL-10F	65	20	5	10	-	-	3.50	0.20
20CC-9LL-6F-10LV	55	20	9	6	10	-	3.50	0.20
25CC-10LL-15LV	50	25	10	-	15	-	3.50	0.20
45GGBFS-10LL	45	-	10	-	-	45	2.75	
CEM III/A (Ref. mix)	50	-	-	-	-	50	2.75	

**Table 3:** Mix design of the two concrete types. PC, CC, LL, LV, and GGBFS are considered as binders for the calculation of the w/b ratio.

Material (kg/m^3^)	CEM II/C-M	CEM III/A
Limestone 0/4	346	349
Recycled aggregate 4/20	889	897
Sea sand 0/2	508	513
CEM I 52.5 N	173	173
Calcined clay	87	-
Limestone powder	35	-
Lava	51	-
GGBFS	-	173
SIKA ViscoCrete 1090	2.5	1.5
Water_free_	173	173
Water_add_	44	44
w/b ratio	0.5	0.5
Flow (EN 12350-5) (mm)	440	440
Air content (EN 12350-7)	1.7%	1.7%

**Table 4:** Mixes for water demand determination on calcined clays and lava.

	Mix	PC	LL	Quartz	CC	LV
ID						
PC + LL + Q		65	10	25	-	-
PC + LL + CC		65	10	-	25	-
PC + LL + LV		65	10	-	-	25

**Table 5:** CO_2_ burdens for the materials used in this study.

Material	Granular Skeleton + Mixing	PC	Clay	Lava	Limestone	GGBFS
kg CO_2_ equivalent/kg	0.0206	0.7166	0.1494	0.0500	0.0026	0.2443

**Table 6:** Modified R3 test results. Heat of dissolution at 3, 7, and 10 days for calcined clay, lava, and reference with inert quartz, and portlandite consumption of lava and calcined clays at the end of the experiment (10 days).

Material	Cumulative Heat Release (J/g SCM)			CH Consumption (g/100 g Solids)
	3 Days	7 Days	10 Days	
Lava	61.6	97.5	137.0	4.36
Calcined clay	220.3	273.4	297.8	13.34
Quartz	19.5	31.0	44.8	-

## Data Availability

The original contributions presented in this study are included in the article. Further inquiries can be directed to the corresponding authors.

## Supplementary Materials

The following supporting information can be downloaded at: https://www.mdpi.com/article/10.3390/ma18215048/s1, Figure S1: TGA and Differential mass loss curve (obtained from TGA measurement) performed on the raw clay (before calcination) for determination of kaolinite content according to [45]; Figure S2: Full particle size distribution curve obtained by optical particle analysis; Figure S3. Freeze thaw tested on 45GGBFS-10LL concrete samples following slightly different testing processes. NaCl 3% refers to the regular protocol described in NBN EN 1339 2003, sat. + NaCl 3% includes saturation of the samples in water before the test using NaCl 3% and NaCl 0% includes the use of tap water instead of the NaCl 3% solution.
