# Supplementary material for: Durability Considerations in Replacing Blast Furnace Slag with Low-Grade Calcined Clay and Natural Pozzolan in Quaternary Cements
Source: Materials (Basel). 2025 Nov 5;18(21):5048. doi: 10.3390/ma18215048 (PMC12608432; doi:10.3390/ma18215048)
Supplement: Supplementary file 1 [file materials-18-05048-s001.zip › materials-3930110-supplementary.pdf]

Supporting information:

TGA was performed in a Netzsch STA 449 Jupiter TGA-DTA Analyzer. The temperature ranged from 20 to 1100 °C at a heating rate of 10 °C/min in an inert nitrogen atmosphere. About 50 mg of raw clay sample was used in TGA experiments. The stepwise method was utilised for the quantification of the kaolinite content for which the range 400-625 °C was adopted (Fig. S1). The range 400 to 625 °C was chosen based on the observations of [47].

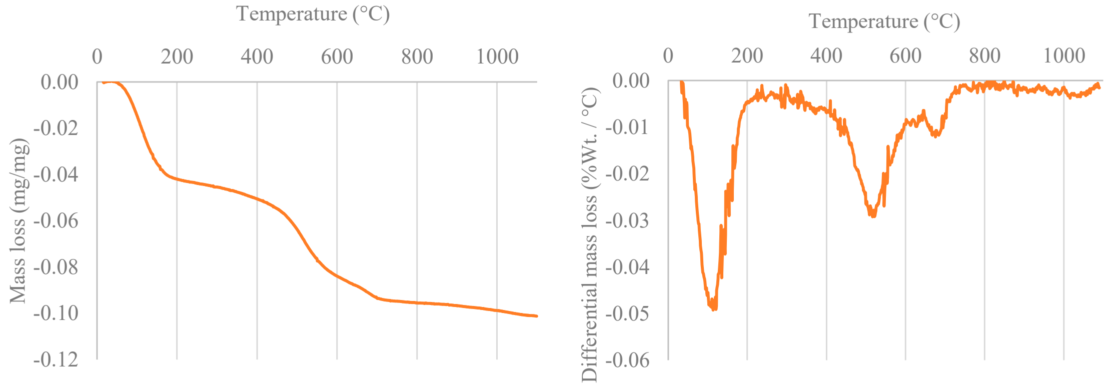

Figure S1. TGA and Differential mass loss curve (obtained from TGA measurement) performed on the raw clay (before calcination).

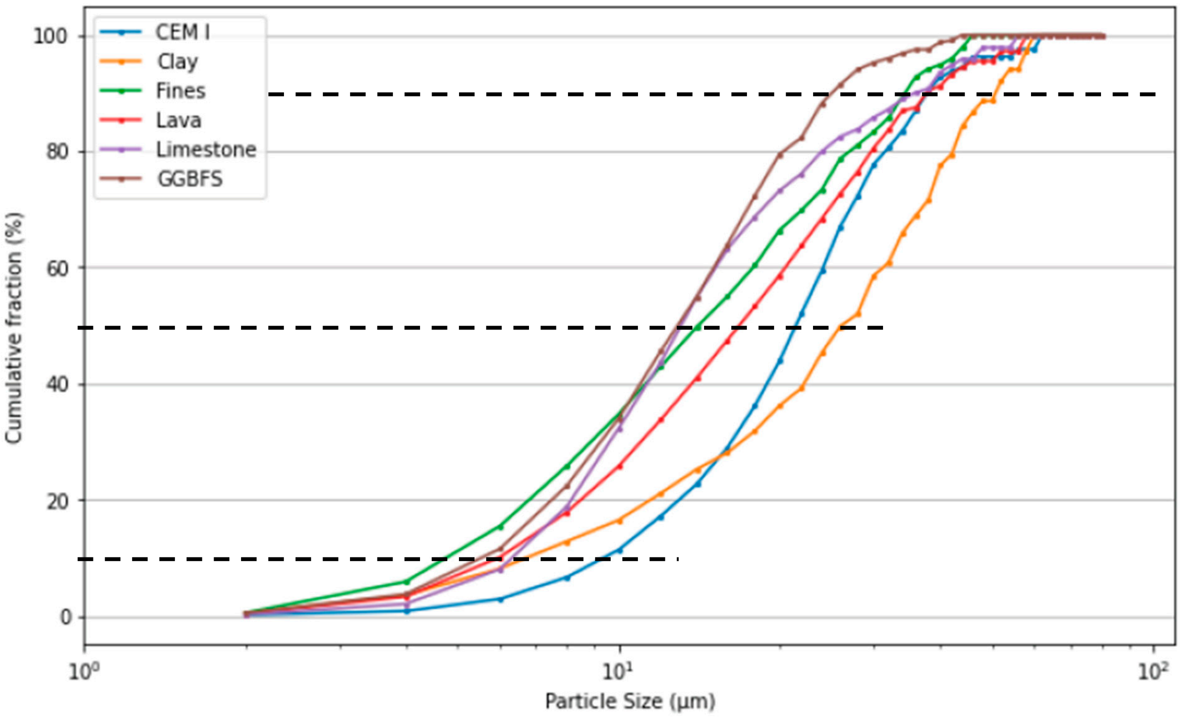

Figure S2. Full particle size distribution curve obtained by optical particle analysis. For each material, the dashed lines indicate the intersections with the D_{v10}, D_{v50}, and D_{v90} values on the horizontal axis (indicated in Table 1).

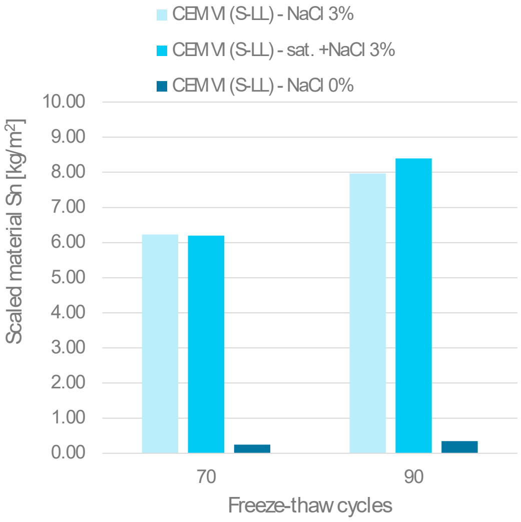

Figure S3. Freeze thaw tested on 45GGBFS-10LL concrete samples following slightly different testing processes. NaCl 3% refers to the regular protocol described in NBN EN 1339 2003, sat. + NaCl 3% includes saturation of the samples in water before the test using NaCl 3% and NaCl 0% includes the use of tap water instead of the NaCl 3% solution..

Figure S3. shows that saturation under vacuum and keeping the samples in water until testing has very limited variations in the freeze-thaw damage. In addition, there is no damage if samples are tested without the inclusion of de-icing salt.
